# Cancer Growth and Invasion Are Increased in the Tight Skin (TSK) Mouse

**DOI:** 10.3390/cancers17182943

**Published:** 2025-09-09

**Authors:** Maria Sol Recouvreux, Barbie Taylor-Harding, Amy C. Rowat, Beth Y. Karlan, Sandra Orsulic

**Affiliations:** 1 Department of Obstetrics and Gynecology, David Geffen School of Medicine, University of California Los Angeles, Los Angeles, CA 90095, USA; barbie.taylor-harding@cshs.org (B.T.-H.); bkarlan@mednet.ucla.edu (B.Y.K.); sorsulic@mednet.ucla.edu (S.O.); 2 Department of Integrative Biology and Physiology, University of California Los Angeles, Los Angeles, CA 90095, USA; rowat@ucla.edu; 3 Jonsson Comprehensive Cancer Center, University of California Los Angeles, Los Angeles, CA 90095, USA; 4 Broad Stem Cell Research Center, University of California Los Angeles, Los Angeles, CA 90095, USA; 5 California NanoSystems Institute, University of California Los Angeles, Los Angeles, CA 90095, USA; 6 Department of Pathology and Laboratory Medicine, David Geffen School of Medicine, University of California Los Angeles, Los Angeles, CA 90095, USA; 7 Department of Veterans Affairs, Greater Los Angeles Healthcare System, Los Angeles, CA 90073, USA

**Keywords:** FBN1, fibrillin, cancer-associated fibroblasts, CAF, myofibroblasts, desmoplasia, cancer invasion, cancer promotion, host response to cancer, systemic sclerosis, scleroderma

## Abstract

**Background**: Patients with systemic sclerosis have a significantly increased incidence of developing various solid malignancies within a few years of systemic sclerosis onset, but the mechanism of tumor promotion is not well understood. The tight skin (TSK) mouse has been a valuable model for investigating systemic sclerosis-related pathologies due to increased extracellular matrix deposition, fibrosis in connective tissues, and altered immune cell activation. Despite the role of extracellular matrix and fibrosis in cancer progression, the potential of the TSK mouse as a model for cancer studies is unexplored. **Methods:** To investigate the impact of the altered microenvironment in TSK mice on cancer progression, we compared the tumor-forming capabilities (by subcutaneous and intraperitoneal injection) in TSK mice and WT mice using syngeneic breast cancer, melanoma, and ovarian cancer cell lines. We used bulk and single-cell RNA sequencing to characterize these tumors and identify the changes in the TSK microenvironment that promote cancer formation. **Results:** In all three cancer types, TSK mice exhibited more invasive subcutaneous tumors in comparison to WT controls, underscoring the role of the TSK subcutaneous microenvironment in promoting cancer progression. Furthermore, the heightened invasiveness of ovarian tumors implanted intraperitoneally suggests that the peritoneal microenvironment in TSK mice also promotes tumor progression. Single-cell RNA sequencing analyses of subcutaneous tumors from TSK and WT mice revealed tumor-specific changes in the composition and phenotype of various cell populations. The most consistent alteration in TSK mice included a higher neutrophil-to-lymphocyte ratio and an enrichment in profibrotic subpopulations of myofibroblasts and macrophages. **Conclusions:** Our research unveils the TSK mouse as a valuable model for studying the intricate connections between systemic sclerosis and cancer

## Simple Summary

People with systemic sclerosis (a chronic disease that causes skin and organ scarring) have a higher risk of developing certain cancers soon after diagnosis, but the reasons remain unclear. To study systemic sclerosis in the laboratory, researchers are using tight skin (TSK) mice, which develop excess scar tissue and immune changes similar to systemic sclerosis. We combined the TSK mouse model with mouse models of skin, breast, and ovarian cancers to test whether the unique environment in TSK mice promotes cancer progression. In all cancer types, TSK mice developed more aggressive tumors. Detailed single-cell analyses showed that TSK tumors had more immune imbalance and more scar-promoting cell types. These findings suggest that the TSK mouse is a powerful tool for uncovering how systemic sclerosis-related tissue changes and immune shifts may fuel cancer growth.

## 1. Introduction

Current therapies for solid malignancies primarily focus on disrupting the growth of actively dividing cancer cells and targeting their genetic modifications. However, it is increasingly evident that the tumor microenvironment has an equally significant influence on tumor progression as a vital source of biomechanical and molecular cues that impact all facets of tumor biology and growth—ranging from proliferation, survival, metabolism, and stem cell fate to therapeutic responses [1]. In particular, the increased presence of cancer-associated fibroblasts (CAFs) and tumor-associated macrophages (TAMs) in solid malignancies has been associated with cancer metastasis and poor patient survival [1,2]. In particular, CAF-secreted extracellular matrix (ECM) has been linked with therapeutic resistance by obstructing the accessibility of chemotherapies and immunotherapies and physically impeding the effective trafficking of functional immune cells to the tumor site [3,4,5,6]. The ECM also serves as a rich reservoir of biologically active molecules, influencing immune cell behavior through the attraction or repulsion of specific immune cell subtypes [7]. Furthermore, elevated levels of enzymes responsible for the formation of collagen cross-links, such as lysyl oxidase [8,9,10], lead to the remodeling of loosely organized connective tissue into linear fiber tracks functioning as conduits that facilitate chemotaxis of tumor cells [11,12]. The increased ECM rigidity of tumors can also impact mechanical signaling and contribute to increased tumor cell invasion/aggressiveness [13,14,15].

A major backbone protein of extracellular microfibrils is Fibrillin-1, encoded by the *FBN1* gene [16,17,18]. In addition to providing structural support, fibrillin regulates the bioavailability of key growth factors and cytokines, such as latent transforming growth factor beta-binding proteins LTBP-1 and LTBP-4, which in turn facilitate the incorporation of the latent form of transforming growth factor beta (TGF-β) into microfibrils [19,20]. Fibrillin-1 is produced by various cell types of mesenchymal origin [21,22,23]. In several cancer types, including ovarian, breast, and pancreatic cancer, elevated fibrillin expression in CAFs has been associated with cancer aggressiveness and poor clinical outcomes [24,25,26]. The exact mechanism by which excess fibrillin might create a conducive microenvironment for cancer progression and metastasis is not fully understood.

The TSK mouse provides a valuable tool for studying the effects of increased fibrillin expression. This model has a mutation that results in an intragenic duplication of exons 17 to 40 of the *Fbn1* gene, which leads to the production of both a normal-sized form (~350 kd) and a mutant oversized form of the Fbn1 protein (~418 kD) [27,28,29]. While homozygous mice die in utero, heterozygous mice are viable but have several health conditions due to the excess of fibrillin [27,28,29]. The TSK mouse model has been used extensively to study systemic sclerosis (scleroderma), as it recapitulates many aspects of systemic sclerosis in humans, including the production of autoantibodies against systemic sclerosis-specific target autoantigens [30,31]. Among other defects that recapitulate cellular features in systemic sclerosis, connective tissues in TSK mice exhibit increased collagen and ECM deposition [32,33], aberrant fibril formation [34], and fibrosis [33]. Notably, collagen levels in the skin and hearts of TSK mice have been found to be higher in males than in females, highlighting the importance of including both genders in comparative studies to ensure unbiased results [35].

Individuals diagnosed with systemic sclerosis exhibit a notably higher occurrence of developing diverse solid malignancies shortly after the onset of systemic sclerosis [36,37,38,39,40,41]. Systemic sclerosis is most frequently linked to the diagnosis of lung and breast carcinomas, which, incidentally, are prevalent in the general adult population as well [36,37,38,39,40,41]. In addition to these common cancer types, systemic sclerosis has also been associated with less prevalent forms, including ovarian, cervical, and kidney carcinomas [42]. Suggested mechanisms for increased risk of cancer in patients with systemic sclerosis include common genetic and environmental risk factors involved in the development of both diseases; acquired genetic damage and immune dysregulation resulting from prolonged immunosuppressive treatments; and increased inflammation and fibrosis in the connective tissue that create a cancer-promoting microenvironment [38]. Here, we tested the hypothesis that cancer progression might be accelerated in TSK mice due to an autochthonous cancer-promoting microenvironment. To our knowledge, this is the first study to investigate tumor growth in the TSK model, and we demonstrate, across three different cancer types, that tumors are more invasive in TSK mice than in WT controls. This finding expands the relevance of the TSK model beyond its established role in fibrosis research and provides new insight into how stromal alterations can drive tumor progression.

## 2. Materials and Methods

### 2.1. Cell Culture

C57BL/6 syngeneic mouse B16F10 melanoma cells [43], EO771 breast cancer cells [44], and SO ovarian cancer cells [45] were grown at 37°C in 5% CO_2_ in DMEM (Corning, Glendale, AZ, USA) supplemented with 10% FBS (MilliporeSigma, Burlington, MA, USA) and 1% penicillin–streptomycin (GIBCO, Thermo Fisher Scientific, Carlsbad, CA, USA) media. Both B16F10 (CRL-6475) melanoma cells and EO771(CRL-3461) Breast cancer cells were acquired from ATCC (Manassas, VA, USA). The SO ovarian cancer cell line (genotype: p53-/-, Hras, myc) was derived from ovarian surface epithelial cells or stem cells from the ovaries of TP53-/- C57BL/6 mice. The TP53-/- cells were transduced with lentiviral vectors containing oncogenes HRAS^V12^ and MYC. This cell line is highly aggressive, forming intraperitoneal tumors and ascites with nearly 100% penetrance. These tumors closely resemble human high-grade, late-stage serous or undifferentiated ovarian carcinoma in their metastatic pattern, with the omentum being the most common site of metastasis.

### 2.2. Mice

All procedures in mice were performed in accordance with the NIH Guide for the Care and Use of Laboratory Animals and approved by the Cedars-Sinai Medical Center Institutional Animal Care and Use Committee (IACUC) or the University of California, Los Angeles (UCLA) Animal Research Committee (ARC). The TSK mutation was maintained by backcrossing mice (Jackson Labs, strain: 014632) with C57BL/6 mice. Both male and female 6- to 10-week-old TSK and WT mice were used for experiments.

### 2.3. Isolation of Dorsal Skin and Bulk RNA Sequencing

Three 8-week-old male TSK mice and three WT male littermates were used for dorsal skin isolation. After shaving the mice, the dorsal skin was removed and processed for RNA isolation using the RNAeasy kit (Qiagen, Germantown, MD, USA). Samples in RT buffer were submitted to the Technology Center for Genomics & Bioinformatics (TCGB) Core at UCLA to be processed for RNA-Seq. Libraries for RNA-Seq were prepared with the KAPA Stranded RNA-Seq Kit (Roche, Montréal, QC, Canada) with the RiboErase Kit (Roche, Montréal, QC, Canada). The workflow consisted of depletion of rRNA by hybridization of complementary DNA oligonucleotides, followed by treatment with RNase H, DNase, and RNA fragmentation. First-strand cDNA synthesis was performed using random priming followed by second-strand synthesis to convert cDNA: RNA hybrids into double-stranded cDNA (dscDNA) and incorporate dUTP into the second cDNA strand. cDNA generation was followed by A-tailing, adaptor ligation, and PCR amplification. Different adaptors were used for multiplexing samples in one lane. Sequencing was performed on an Illumina HiSeq 3000 for an SE 1 × 50 run. Data quality check was performed on an Illumina SAV. Demultiplexing was performed with the Illumina Bcl2fastq (version 2.19.1.403 software). The alignment was performed using STAR [46] with mouse reference genome mm10. The mm10—Ensembl Transcripts release 100 gtf was used for gene feature annotation. The Partek Flow software (version 11.0), was used to determine read counts normalized by CPM and to perform data analysis.

### 2.4. Isolation of Dorsal Skin and Single-Cell RNA Sequencing

Dorsal skin collected from 3 TSK mice and 3 WT littermates was pooled and minced into 1 mm pieces. Tissues were further dissociated by enzymatic digestion by rotating at 37 °C for 60 min with 10 mg/mL collagenase I, 5 mg/mL collagenase IV, 10 mg/mL collagenase D, and 5 mg/mL DNAse I (Selleckchem, Houston, TX, USA) in DPBS (GIBCO, Thermo Fisher Scientific, Carlsbad, CA, USA). After dissociation, the suspension was strained with a 70-micron strainer (Greiner Bio-one, Monroe, NC, USA) followed by 8 min centrifugation at 300 g. Red blood lysis buffer (Thermo Fisher Scientific, Carlsbad, CA, USA) was used, followed by dead cell removal (STEMCELL Technologies, Seattle, WA, USA), to deplete the samples of erythrocytes and dead cells, respectively, according to the manufacturers’ instructions. Purified cells were resuspended in 0.04% BSA-PBS solution at a concentration of 700–1000 cells/microliter and submitted for library preparation at the UCLA TCGB Core. The Partek Flow software (version 11.0) was used for thresholding and single-cell RNA sequencing data analysis. Cells were removed according to the following criteria: (1) cells had fewer than 200 genes or more than 10,000 genes; (2) cells had fewer than 500 unique molecular identifiers (UMIs) or over 10,000 UMIs; and (3) cells had more than 10% mitochondrial UMI counts.

### 2.5. Syngeneic Tumor Growth in Mice and Single-Cell RNA Sequencing Analysis

TSK and WT mice were injected subcutaneously or intraperitoneally with syngeneic melanoma, breast cancer, or ovarian cancer cell lines (1 × 10^5^ to 2 × 10^6^ cells per injection, as indicated for each experiment), and tumor growth was monitored for 14–18 days depending on the experiment. Subcutaneous tumors obtained from each mouse were divided in half and pooled for IHC or single-cell RNAseq analysis. Preparation for single-cell RNA sequencing and single-cell RNA sequencing analyses was accomplished as previously described [45]. The Partek Flow software (version 11.0) was used for thresholding and single-cell RNA sequencing data analysis. Cells were removed according to the following criteria: (1) cells had fewer than 200 genes or more than 10,000 genes; (2) cells had fewer than 500 UMIs or over 10,000 UMIs; and (3) cells had more than 10% mitochondrial UMI counts.

### 2.6. Immunohistochemistry and Antibodies

Immunohistochemistry on formalin-fixed paraffin-embedded tissue sections was performed by the UCLA Translational Pathology Core Laboratory (TPCL) using the Leica Bond RX processor based on Protocol F using the Bond Polymer Refine Detection kit (Cat#DS9800, Leica Biosystems, Nussloch, Germany); ER2 buffer (BOND Epitope Retrieval Solution 2,Cat# AR9640, Leica Biosystems, Nussloch, German)y; DakoCytomation Envision System Labelled Polymer HRP anti-rabbit (Agilent, Santa Clara, CA, USA, K4003); and antibodies against LCN2 (NGAL Antibody, PA5-79590, Invitrogen, Rockford, IL, USA), CD3 (DAKO A0452, Agilent, Santa Clara, CA, USA), and NKR (Anti-NKR-P1C antibody [EPR22990-31], Abcam, Waltham, MA, USA). Hematoxylin (MilliporeSigma, Burlington, MA, USA) was used as the counterstain. Trichrome staining was also performed by the TPCL core using standard protocols.

### 2.7. Pathology Image Analyses

The coverslipped slides were scanned at 20× magnification with the Aperio ScanScope AT slide scanner (Aperio, Vista, CA, USA). Tumor invasiveness in H&E and Masson’s trichrome slides was analyzed by overlaying the image with 250 µm square tiles and manually scoring each tile as positive if it contained noncancer tissue encircled by cancer cells. Noncancer tissues included fibroblasts; muscle cells; adipose cells; blood vessels; and peritoneal organ tissues, such as the spleen, liver, and intestine. Each tumor nodule from WT and TSK mice was individually analyzed, with each contributing one data point to the percentage of positive tiles graph.

Neutrophil-to-lymphocyte ratio (LCN2^+^ cells: (CD3^+^ cells and NKR^+^ cells)) was quantified in consecutive immunostained sections using Qpath’s “positive cell detection” function [47]. For LCN2 staining, we applied a high-intensity threshold to selectively detect only strongly positive cells, minimizing background from other cell types such as fibroblasts. The accuracy of this selection was confirmed by verifying the morphology of the positively stained cells.

### 2.8. Statistical Analysis

Data were expressed as means ±  SEM of three or more independent experiments unless otherwise stated. Statistical analyses were performed using GraphPad Prism (version 9.0; GraphPad Software). Statistically significant data were assessed by unpaired Student’s *t*-tests unless otherwise noted. Intergroup differences were considered statistically significant at *p* ≤ 0.05.

## 3. Results

### 3.1. Dorsal Skin Fibroblasts from TSK Mice Exhibit Features of CAFs

**Figure 1:**
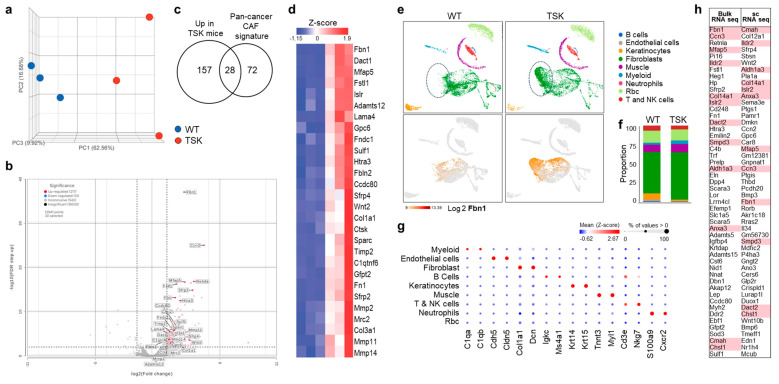
**Bulk and single-cell RNA sequencing comparison of dorsal skins isolated from WT mice and TSK littermates.** (**a**) Principal component analysis (PCA) for bulk skin expression profiles of 3 WT mice and 3 TSK littermates. (**b**) Volcano plot of differentially expressed genes between bulk skin from WT and TSK mice (DESeq2, fold change > 2.5, *p* < 0.01, False Discovery Rate (FDR) correction *p* < 0.01). (**c**) Venn diagram depicting the number of overlapping upregulated genes in the bulk skin of TSK mice and a pancancer gene signature of cancer-associated fibroblasts (CAFs). (**d**) Heatmap of normalized expression levels of genes upregulated in dorsal skin of TSK mice that overlap with the human pancancer gene signature of activated CAFs. (**e**) Upper panels: Unsupervised graph-based clustering of single-cell suspensions of dorsal skins isolated from 3 WT mice and 3 TSK littermates visualized by a uniform manifold approximation and projection (UMAP) plot. Each cluster is color-coded according to the cell type annotation, and the relative proportion of each cell cluster in WT and TSK samples is shown. Lower panels: UMAP depicting FBN1 expression in fibroblasts. (**f**) Histogram indicating the proportion of individual cell types in skin from WT and TSK mice. (**g**) Dot plot showing the expression of representative cell-type-specific markers across different cell clusters. The color intensity reflects the average gene expression, and the size indicates the percentage of cells expressing the gene within that cell type. (**h**) Fifty most significantly differentially expressed genes identified by bulk and single-cell RNA sequencing analyses. Genes identified by both methods are highlighted in pink.

The most striking feature of TSK mice is the structural alteration of dorsal skin tissue, giving it a firm, tight appearance [27,28,29]. In solid malignancies, CAF-secreted ECM is the primary driver of tumor stiffness [1]. To assess the potential similarity of TSK fibroblasts to tumor-promoting CAFs, we compared the expression profiles of shaved dorsal skin isolated from three TSK mice and three WT littermates using bulk and single-cell RNA sequencing expression profiles. Principal component analysis of bulk tissue RNA transcripts revealed clear separation between TSK and WT skin (Figure 1a). Differential gene expression analysis (DESeq2, fold change > 2.5, *p* < 0.01, False Discovery Rate (FDR) correction) resulted in 185 genes upregulated in TSK skin and 10 genes upregulated in WT skin (Table S1). As expected, Fbn1 was one of the highly upregulated genes in dorsal skin from TSK mice (Figure 1b and Table S1). Of the 185 genes upregulated in TSK skin, 28 overlapped with the 100-gene pancancer signature of activated CAFs [48] (Figure 1c,d).

To identify the cell types contributing to altered gene expression in the skin of TSK mice, we performed a single-cell RNA sequencing analysis on disaggregated dorsal skins from TSK mice and WT littermates. Graph-based clustering of cells following uniform manifold approximation and projection (UMAP) analysis identified nine distinct cell types, most of which were present in similar proportions and exhibited comparable phenotypes between WT and TSK littermates (Figure 1e,f). Cell identities were determined based on the canonical marker genes (Figure 1g) and the representation of differentially expressed genes in each cluster in the ImmGen database [49]. TSK mice had fewer keratinocytes (Figure 1e), which were enriched for genes identified in bulk skin RNA sequencing analysis, including Fbn1, Mfap5, and Fstl1 (Figure S1). The most striking difference between WT and TSK mice was in fibroblast phenotypes, with TSK mice exhibiting a distinct fibroblast population absent in WT mice (Figure 1e, dotted ellipse). This fibroblast population was characterized by high expression of Fbn1 (Figure 1e, bottom panels). Besides Fbn1, differential gene expression analysis (DESeq2, fold change > 2.5, *p* < 0.01, FDR correction) identified an additional 123 genes upregulated in TSK fibroblasts and 253 genes upregulated in WT fibroblasts (Table S2). Overall, both bulk and single-cell RNA sequencing revealed overlapping sets of upregulated genes in TSK skin, reinforcing the reliability of these methods (Figure 1h).

Several of the genes found to be significantly upregulated in TSK fibroblasts by both methods have previously been linked to systemic sclerosis, other autoimmune diseases, and cancer progression. Notably, cellular communication network factor 3 (Ccn3), a member of the CCN (Cyr61/Ctgf/Nov) family of developmental regulators (Figure 1h), was previously identified as one of the most highly upregulated genes in TSK fibroblasts [50]. Ccn3 has been implicated in multiple immune-related diseases, including systemic sclerosis [51], and is known to promote invasion and metastasis in various solid cancers, such as breast, prostate, bladder, and melanoma [52,53]. Our identification of increased stromal microfibrillar-associated protein 5 (Mfap5, also known as MAGP-2) expression in TSK skin (Figure 1b) aligns with prior reports of elevated Mfap5 levels in the dermises of both TSK mice and patients with systemic sclerosis [54]. Mfap5 is also upregulated in cancer-associated fibroblasts across multiple solid tumors, reinforcing its role in fibrosis and tumor progression [55]. Additionally, we found increased expression of immunoglobulin-like domain-containing receptor 2 (Ildr2) (Figure 1h), a gene involved in immune tolerance and T cell regulation in autoimmune diseases and cancer [56,57]. The upregulation of these genes in TSK fibroblasts further supports their shared pathogenic mechanisms in fibrosis, immune dysregulation, and cancer. Since dorsal skin fibroblasts in TSK mice exhibit characteristics resembling activated CAFs, we hypothesized that cancer growth would be enhanced in TSK mice. We tested this hypothesis using syngeneic models of melanoma, breast cancer, and ovarian cancer.

### 3.2. Melanoma

**Figure 2:**
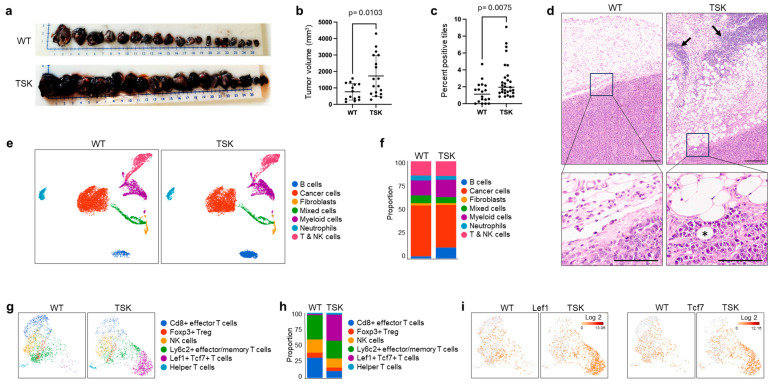
**Increased invasiveness and growth of subcutaneous melanoma in TSK mice.** (**a**) Male TSK mice (*n* = 10) and WT littermates (WT, *n* = 11) were subcutaneously injected with the syngeneic mouse melanoma B16F10 cell line in the flank area on both sides of the body (2 × 10^6^ cells in each flank). The subcutaneous tumors were harvested after 14 days. (**b**) Tumor volume in WT and TSK mice. Unpaired *t*-test. (**c**) Percentage of 250 µm^2^ tiles overlayed on digitized tumor sections of H&E images that contained muscle cells, adipose cells, and/or vasculature surrounded by cancer cells. Unpaired *t*-test. (**d**) Representative H&E-stained sections of subcutaneous tumors. The arrows in the upper panels indicate immune cell infiltrates. Scale bars, 200 µm. The lower panels are magnified images of the square areas in the upper panels. The asterisk indicates an adipocyte engulfed by cancer cells. Scale bars, 10 µm. (**e**) Unsupervised graph-based clustering of single-cell suspensions of tumors isolated from WT and TSK mice, visualized by UMAP plot. Each cluster is color-coded according to the cell type annotation. (**f**) Histogram showing the proportions of individual cell types in tumors from WT and TSK mice. (**g**) UMAP plot of reclustered T cells. Each cluster is color-coded based on differential gene expression in individual immune cell subsets. (**h**) Histogram showing the proportions of individual T cell types. (**i**) Heatmap overlay of log 2 expression of Lef1 and Tcf7 in T cell clusters.

The syngeneic melanoma cancer cell line B16F10 [43] was injected subcutaneously into both hind flanks of male WT (11) and TSK (10) mice. After 14 days, subcutaneous tumors were harvested (Figure 2a). The tumors from TSK mice were larger than the tumors from WT mice (Figure 2a,b). The most noticeable difference, though difficult to quantify, was that the tumors in TSK mice were more deeply embedded in the subcutaneous mesenchyme and peritoneal wall tissues and, therefore, harder to resect. H&E staining of tumor sections confirmed the pronounced invasion of cancer cells into the mesenchyme and adipose tissue in TSK mice (Figure 2c,d and Figure S3). Single-cell RNA sequencing of melanoma tumors revealed an increased proportion of B cells/plasma cells in TSK mice (Figure 2e,f); however, closer inspection of tumor sections revealed that the immune infiltrates were located primarily in the mesenchyme surrounding the tumor tissue (Figure 2d). Reclustering of lymphocytes revealed that subsets of T cells were differentially represented in WT and TSK mice, with a decreased proportion of helper T cells and an increased proportion of Lef1 + Tcf7 + T cells (Figure g–i).

### 3.3. Breast Cancer

**Figure 3:**
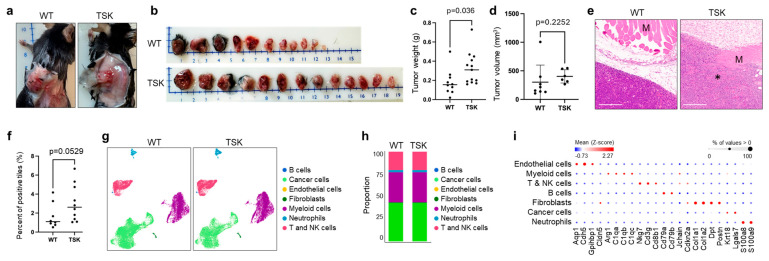
**Increased invasiveness of subcutaneous breast cancer in TSK mice.** Female TSK mice (*n* = 10) and WT littermates (*n* = 11) were subcutaneously injected with the EO771 syngeneic mouse breast cancer cell line in the flank area on both sides of the body (2 × 10^6^ cells in each flank). (**a**) Representative picture of a subcutaneous tumor from WT and TSK mice. (**b**) The subcutaneous tumors were harvested after 18 days. (**c**) Tumor weight in WT and TSK mice. Unpaired *t*-test. (**d**) Tumor volume in WT and TSK mice. Unpaired *t*-test. (**e**) Representative H&E-stained sections of subcutaneous tumors. The asterisk indicates tumor cell invasion into the muscle tissue (M). Scale bar, 300 µm. (**f**) Percentage of 250 µm^2^ tiles overlayed on digitized tumor sections of H&E images that contained muscle cells, adipose cells, and/or vasculature surrounded by cancer cells. (**g**) Unsupervised graph-based clustering of single-cell suspensions of tumors isolated from WT and TSK mice, visualized by UMAP plot. Each cluster is color-coded according to the cell type annotation. (**h**) Histogram showing the proportions of individual cell types in tumors from WT and TSK mice. (**i**) Dot plot showing the expression of representative cell-type-specific markers across different cell clusters. The color intensity reflects the average gene expression, and the size indicates the percentage of cells expressing the gene within that cell type.

The syngeneic breast cancer cell line EO771 [44] was injected subcutaneously into both hind flanks of female WT and TSK mice. After 18 days, subcutaneous tumors were harvested. Mirroring the pattern observed with the melanoma cancer cell line, tumors in TSK mice were more deeply embedded in the subcutaneous mesenchyme (Figure 3a). The weight of the resected tumors was slightly higher in TSK mice, while the volume was not significantly different (Figure 3b,d). H&E staining of tumor sections and computational image analyses of tiles containing muscle, adipose cells, and blood vessels surrounded by cancer cells showed a clear trend of increased cancer cell invasion into the mesenchyme and muscle tissue in TSK mice, although it did not achieve statistical significance (Figure 3e,f). Single-cell RNA sequencing analysis of breast tumors did not indicate significant differences in the proportions or phenotypes of various cell subsets (Figure 3g–i). Overall, the primary distinction between breast EO771 tumors in WT and TSK mice was the extent of tumor embedding within the mesenchyme, as observed during tumor resection.

### 3.4. Ovarian Cancer

In our previous covariate-adjusted single-gene analyses of 3769 ovarian cancer patients, FBN1 was identified as the top gene associated with poor overall survival [24], suggesting that it might have a functional role in ovarian cancer progression. FBN1 is enriched in the mesenchymal (desmoplastic) molecular subtype of ovarian cancer (Figure S4a) and is primarily expressed in ovarian fibroblasts and the fibroblast-secreted ECM (Figure S4b). FBN1 is expressed at higher levels in the cancer stroma than in normal ovarian stroma (Figure S4c). In the Cancer Genome Atlas (TCGA) and Australian Ovarian Cancer Study (AOCS) transcriptome datasets, the 185 gene signature upregulated in the dorsal skin of TSK mice (Table S1) were enriched in the mesenchymal molecular subtype (Figure S5) and in cases characterized by residual disease after primary debulking surgery, venous and lymphatic invasion, and ovarian cancer metastasis (Figure S6).

**Figure 4:**
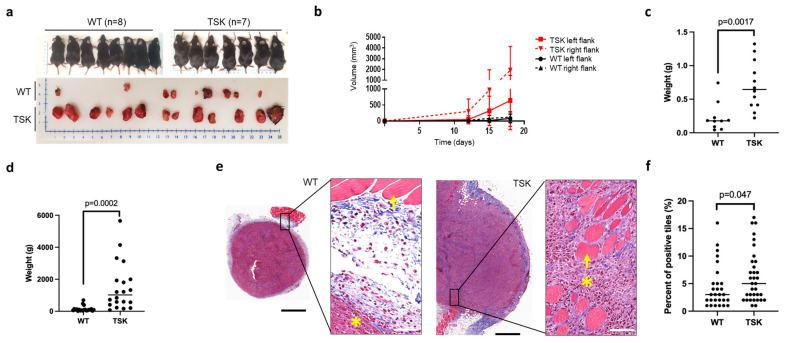
**Accelerated progression of subcutaneous ovarian cancer in TSK mice.** (**a**) Male TSK (*n* = 7) and WT (*n* = 8) mice were subcutaneously injected in both flanks with 1 × 10^6^ SO syngeneic mouse ovarian cancer cells per site. Tumors were harvested on day 18, when ulceration began in TSK mice. (**b**) Subcutaneous tumor volume measured over time. (**c**) Combined wet tumor weight from both flanks presented as a single value per mouse (paired *t*-test). (**d**) The same experimental design was repeated in female TSK (*n* = 13) and WT (*n* = 11) mice. Combined wet tumor weight from both flanks is presented as a single value per mouse (paired *t*-test). (**e**) Representative Masson’s trichrome-stained sections of subcutaneous ovarian cancer nodules. Asterisks indicate tumors, while arrows indicate muscle. Scale bars: 600 µm and 60 µm in low- and high-magnification images, respectively. (**f**) Percentage of 250 µm^2^ tiles overlayed on digitized tumor sections of Masson’s trichrome-stained images that contained muscle cells, adipose cells, and/or vasculature surrounded by cancer cells. Unpaired *t*-test.

The syngeneic ovarian cancer cell line SO (p53-/-, myc, H-ras) was injected under the skin of both hind flanks of TSK and WT mice. Both male and female TSK mice exhibited larger subcutaneous tumors than their WT littermates (Figure 4a–d). Additionally, the subcutaneous tumors in TSK mice were more firmly anchored in the surrounding mesenchyme and frequently engulfed the muscle layer of the skin (Figure 4e,f and Figure S7), following the pattern observed in subcutaneous melanoma and breast tumors.

**Figure 5:**
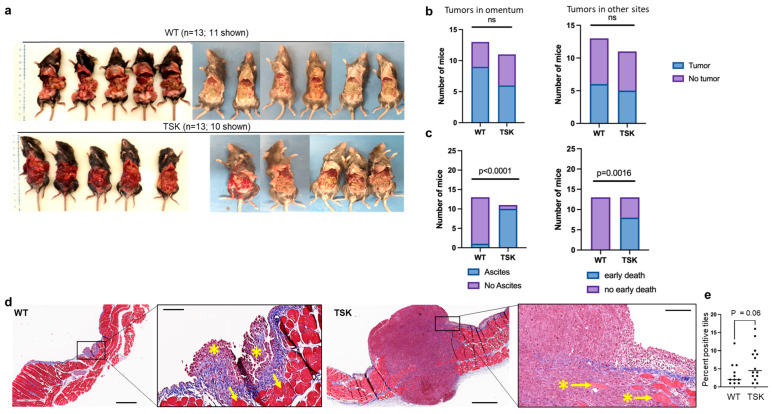
**Accelerated progression of intraperitoneal ovarian cancer in TSK mice.** (**a**) Results from two independent experiments in female mice with a total of 13 WT and 13 TSK mice. Mice were intraperitoneally injected with 1 × 10^6^ SO syngeneic mouse ovarian cancer cells. At necropsy, ascites was present in TSK mice but absent in WT mice. Both groups of mice exhibited similar tumor burden on the omentum and other sites. (**b**) Histograms showing the presence of tumors in the omentum (left) and other sites (right), Chi-square (χ^2^) test. (**c**) Histograms showing the presence of ascites (left) and early onset of death (right) as a measurement of disease severity, Chi-square (χ^2^) test. (**d**) Representative Masson’s trichrome-stained sections of intraperitoneal tumors from WT and TSK mice. Scale bars: 600 µm and 100 µm in low- and high-magnification images, respectively. Asterisks indicate tumors, while arrows indicate muscle. (**e**) Percentage of 250 µm^2^ tiles overlayed on digitized tumor sections of Masson’s trichrome-stained images that contained muscle cells, adipose cells, vasculature, and peritoneal organ tissues surrounded by cancer cells, unpaired *t*-test.

To assess whether the intraperitoneal microenvironment in TSK mice also alters tumor progression, female TSK and WT mice were intraperitoneally injected with SO ovarian cancer cells. A total of 13 WT and 13 TSK mice were injected with SO cells in two different experiments (Figure 5a). In both cases, TSK mice developed tumors earlier, and 5 out of 13 TSK mice died before the stipulated endpoint. At day 16, the mice were euthanized, and tumor burden was assessed by the presence of ascites, tumor nodules on the omentum, tumor nodules at other sites, and the early onset of death. There was no difference in the presence of tumor nodules in the omentum or other sites, but TSK mice exhibited the presence of ascites and early onset of death (Figure 5c). Histological analysis revealed that the tumors in TSK mice were typically more invasive than in WT mice (Figure 5d,e and Figure S7).

**Figure 6:**
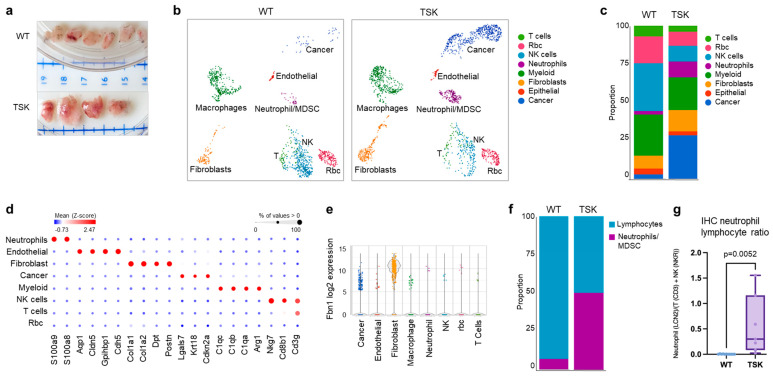
Single-cell RNA sequencing-based tumor microenvironment analysis of syngeneic subcutaneous ovarian tumors from WT and TSK mice. (**a**) Picture of tumors collected from 4 WT mice (top) and 4 TSK mice (bottom) used for single-cell RNA sequencing analysis. (**b**) Unsupervised graph-based clustering of single-cell suspensions of tumors isolated from WT and TSK mice, visualized by UMAP plot. Each cluster is color-coded according to the cell type annotation. (**c**) Histogram showing the proportions of individual cell types in tumors from WT and TSK mice. (**d**) Dot plot showing the expression of representative cell-type-specific markers across different cell clusters. The color intensity reflects the average gene expression, and the size indicates the percentage of cells expressing the gene within that cell type. (**e**) Violin plots of log 2-transformed Fbn1 expression in different cell clusters. (**f**) Histogram showing the proportion of T and NK cells vs. neutrophils/MDSC in tumors from WT and TSK mice. (**g**) Neutrophil-to-lymphocyte ratio calculated from immunohistochemical staining of tumor sections with antibodies specific for T cells, NK cells, and neutrophils. Unpaired *t*-test. Rbc, red blood cells.

To better understand the differences in cell populations in TSK and WT mice, we conducted a single-cell RNA sequencing analysis of subcutaneous tumors isolated from four TSK and four WT mice (Figure 6a). Graph-based clustering identified eight distinct cell clusters (Figure 6b,c). Fbn1 was primarily expressed in fibroblasts (Figure 6d). WT and TSK mice exhibited different proportions of individual cell clusters. WT tumors were enriched in cell clusters representing T and NK cells, while TSK tumors were enriched in cell clusters representing cancer cells, macrophages, neutrophils/MDSCs, and fibroblasts (Figure 6e and Figure S8). Tumors from TSK mice also had a higher neutrophil-to-lymphocyte ratio (Figure 6f), which was validated by immunohistochemical analysis of tumor sections stained with antibodies specific for T cells (CD3), NK cells (NKR), and neutrophils (LCN2) (Figure S9 and Figure 6g and Table S3).

**Figure 7:**
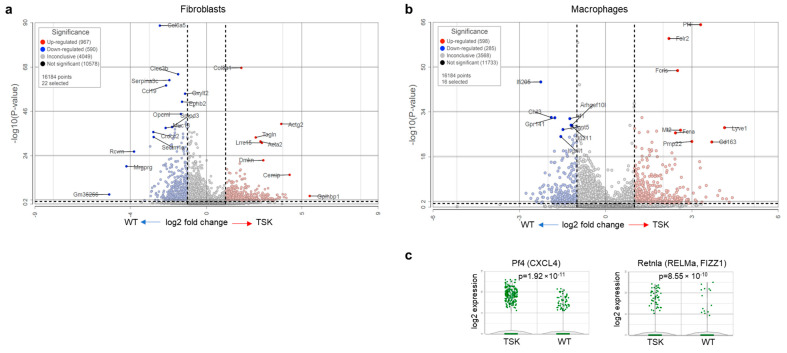
**Fibroblast and macrophage expression comparison of WT and TSK ovarian tumors.** (**a**) Volcano plot representing differential gene expression (ANOVA) in fibroblasts between WT and TSK mice. (**b**) Volcano plot representing differential gene expression (ANOVA) in macrophages between WT and TSK mice. (**c**) Violin plot showing PF4 (left) and Retnla (right) expression in macrophages between WT and TSK mice. ANOVA.

The fibroblast cluster in tumors from TSK mice was enriched in collagen type VIII alpha 1 chain (Col8a1) and myofibroblast markers smooth muscle actin gamma 2 (Actg2), smooth muscle actin alpha (Acta2), transgelin (Tagln), and leucine-rich repeat containing 15 (Lrcc15) (Figure 7a). The macrophage cluster in TSK tumors was significantly rich in platelet-derived factor 4 (Pf4, also known as Cxcl4) (Figure 7b,c), an established biomarker in systemic sclerosis [58,59,60]. Pf4 functions as a chemoattractant for monocytes, neutrophils, and fibroblasts and has a role in triggering monocytes and macrophages to facilitate myofibroblast activation and fibrosis [61,62,63]. The macrophage cluster in TSK tumors also exhibited the higher expression of a member of a cysteine-rich secreted protein family, resistin-like alpha (Retnla, also known as RELMa or FIZZ1) (Figure 7b,c). It was recently shown that Retnla is transiently expressed in monocytes upon their entry into tissues and transition into tissue-resident macrophages [64]. Retnla expression is typically observed in tissue inflammation and fibrosis and is associated with increased expression of Acta2 and fibrillar collagens in fibroblasts [65]. Retnla knockout mice were shown to be resistant to bleomycin-induced lung fibrosis, while Retnla overexpression exacerbated fibrosis [65]. In our analysis, Retnla was one of the top overexpressed genes in intact dorsal skin of TSK mice (Figure 1b), suggesting that its elevated expression may not be associated with bleomycin-induced tissue injury or tumor growth but is innate to the connective tissues of TSK mice, where it might facilitate fibrosis. Notably, Pf4 and Retnla were also upregulated in melanoma and breast cancer macrophages in TSK mice compared to tumor macrophages in WT littermates, although the overexpression did not reach statistical significance (Figure S10).

## 4. Discussion

Systemic sclerosis is an autoimmune disease characterized by connective tissue fibrosis and vasculopathy [66]. The severe morbidity and high mortality associated with systemic sclerosis, coupled with the lack of any disease-modifying therapy, highlight the urgent need for innovative research. Patients with systemic sclerosis have an increased rate of multiple cancer types compared to the general population [36,37,38,39,40,41,42]. The TSK mouse model, recognized for its relevance in the studies of systemic sclerosis, has thus far remained unexplored in the realm of cancer. The findings of our study shed light on the potential of the TSK mouse model in understanding the complex interplay between systemic sclerosis and cancer.

The mechanism by which the microenvironment in TSK mice promotes tumor progression is poorly understood. In addition to fibrosis, TSK mice exhibit other features of systemic sclerosis, including altered immune cell activation [67,68]. Thus, multiple mechanisms could contribute to accelerated tumor progression in TSK mice. To better understand the differences in cell populations in WT and TSK mice, we conducted bulk tissue and single-cell RNA sequencing analyses of dorsal skin tissue. These analyses highlight a distinct gene expression profile in the dorsal skin of TSK mice, characterized by an enrichment of genes associated with cancer aggressiveness and desmoplasia. This observation aligns with the increased incidence of solid malignancies in patients with systemic sclerosis. Importantly, our study demonstrates that the microenvironment in TSK mice promotes cancer progression, as evidenced by larger and more invasive tumors in comparison to WT controls. We showed that intact dorsal skin from TSK mice exhibits higher expression levels of key genes involved in CAF activation and cancer progression. Increased MFAP5 expression in CAFs has been associated with tumor aggressiveness and poor survival in several cancer types, including ovarian, pancreatic, colorectal, and breast cancer [69,70,71,72,73]. Antibody-mediated inhibition of Mfap5^high^ CAFs was effective in reducing fibrosis and enhancing chemosensitivity in ovarian and pancreatic cancer mouse models [71,74]. Another gene upregulated in our RNA sequencing analysis of dorsal skin samples and single-cell RNA sequencing of TAMs from TSK mice was Retnla. Multiple studies have confirmed the crucial roles of Retnla in lung and dermal fibrosis [65,75]. These findings open avenues for further exploration of targeted therapies aimed at these molecular components. For instance, the potential targeting of Mfap5^high^ CAFs and Retnla^high^ TAMs may offer novel strategies to alleviate symptoms in systemic sclerosis and reduce the frequency of cancer development in affected individuals.

To delve deeper into the molecular origins of increased tumor growth and invasion in TSK mice, we performed a single-cell RNA sequencing analysis of melanoma, breast cancer, and ovarian cancer implanted under the skin of TSK and WT mice. This approach revealed a higher neutrophil-to-lymphocyte ratio in all tumors, with the most significant difference observed in ovarian cancers in TSK mice. The neutrophil-to-lymphocyte ratio is linked to disease severity in systemic sclerosis patients [76,77]. Moreover, identifying Lrcc15 as an enriched gene in TSK CAFs in ovarian cancer raises intriguing possibilities for therapeutic interventions. LRRC15^+^ CAFs have been associated with tumor promotion, immunotherapy resistance, and inhibition of CD8^+^ T cell function [78,79]. Selective depletion of LRRC15 + CAFs has shown promise in slowing tumor growth, reducing metastasis, and enhancing the efficacy of immunotherapy [79,80]. The advancement of therapies aiming to reinstate the normal fibroblast equilibrium by decreasing the presence of LRRC15^+^ myofibroblasts associated with disease could potentially enhance patient survival and improve the efficacy of immunotherapy. LRRC15-antibody targeted drug conjugate ABBV-085 has been developed to target LRCC15^+^ stromal desmoplasia in sarcomas and carcinomas, including ovarian cancer [81,82]. The insights from this study pave the way for future investigations into the therapeutic potential of selectively targeting LRRC15 in the context of systemic sclerosis and cancer.

Furthermore, our study highlights the role of Pf4, a biomarker in systemic sclerosis [58,59,60], as a significantly upregulated gene in macrophages from TSK tumors. Pf4 is known for its function in triggering monocytes and macrophages to facilitate myofibroblast activation and fibrosis in systemic sclerosis and cancer [61,62,63]. Pf4-/- mice are protected from bleomycin-induced fibrosis in multiple organs, including the skin, lungs, and heart. Blocking Pf4 with a monoclonal antibody completely suppresses the increase in dermal thickness after bleomycin treatment, while Pf4 overexpression aggravates bleomycin-induced skin fibrosis [63]. Thus, targeted inhibition of Pf4 may present a viable strategy for mitigating fibrosis in both systemic sclerosis and cancer.

While the TSK model has provided valuable insights into fibrosis and extracellular matrix biology, it is important to note its limitations. TSK mice do not fully recapitulate all aspects of systemic sclerosis, particularly the heterogeneity and complexity of disease observed in patients. Clinical systemic sclerosis encompasses diverse manifestations, including variable degrees of skin, vascular, and internal organ involvement, which are not reflected in the TSK model. Another limitation of this study is that we did not consider the immune cell activity, but since all tumor studies were performed in syngeneic mice, the likelihood of an immune-mediated rejection response is low. It is important to note that in this context, the immune contribution is expected to be minimal, particularly at later stages of tumor growth when tumors are fully established. Thus, findings from TSK mice should be interpreted within the context of these limitations, with the recognition that they model selected features of the disease rather than its full spectrum.

The primary focus of this study was to examine the influence of the TSK stromal microenvironment on tumor growth. Although direct therapeutic or diagnostic implications are beyond the scope of this work, our findings underscore the role of stromal alterations in modulating tumor invasiveness, providing a foundation to explore translational applications.

Future studies could extend these findings to other epithelial cancers, as scleroderma patients are predisposed to a broad range of tumor types. Additionally, it will be important to investigate how the TSK stromal background influences the growth and progression of non-epithelial malignancies, including sarcomas and hematologic (liquid) tumors. Such studies could further elucidate the generalizability of stromal effects on tumor biology and provide insight into disease-specific vulnerabilities across cancer types.

## 5. Conclusions

Our research unveils the TSK mouse as a valuable model for studying the intricate connections between systemic sclerosis and cancer. The identified molecular and cellular changes in the TSK microenvironment provide a foundation for future investigations, aiming to unravel the mechanisms underlying enhanced tumorigenicity in this model. Ultimately, these insights hold the potential for developing innovative therapeutic strategies targeting key players in the systemic sclerosis–cancer axis.

## Data Availability

Data obtained through bulk RNA sequencing and single-cell RNA sequencing of skin and tumor tissues have been deposited in the Gene Expression Omnibus (GEO) under the following accession numbers: bulk RNA-seq skin (GSE255493), single-cell RNA-seq skin (GSE292228), single-cell RNA-seq melanoma (GSE292227), single-cell RNA-seq breast cancer (GSE292229), and single-cell RNA-seq ovarian cancer (GSE255492).

## Supplementary Materials

The following supporting information can be downloaded at https://www.mdpi.com/article/10.3390/cancers17182943/s1: Figure S1: Differentially expressed genes in keratinocytes from dorsal skins of WT and TSK mice. Figure S2: Quantification of tumor invasiveness. Figure S3: Expression of canonical cell type markers in subcutaneous melanoma B16F10 tumors analyzed by single-cell RNA sequencing. Figure S4: Fibrillin expression in human ovarian cancer. Figure S5: Gene signature enriched in the skin of TSK mice is associated with the mesenchymal (c1) molecular subtype of ovarian cancer. Figure S6: Gene signature enriched in the skin of TSK mice is associated with ovarian cancer invasion, metastasis, and suboptimal surgical debulking. Figure S7: Quantification of tumor invasiveness. Figure S8: Ovarian tumors from WT mice are enriched for T cells and NK cells, while ovarian tumors from TSK mice are enriched for neutrophils. Figure S9: Immunohistochemical detection of T cells (CD3 antibody), NK cells (NKR-P1 antibody), and neutrophils (LCN2 antibody). Figure S10: Upregulation of Pf4 and Retnla expression in myeloid cells in melanoma and breast tumors in TSK mice. Table S1: Differentially expressed genes in TSK and WT dorsal skin, bulk RNA-seq. Table S2: Differentially expressed genes in TSK and WT dorsal skin, single-cell RNA seq. Table S3. Associated to Figure 6g. Absolute number of positive cells for CD3, NKR, and LCN2.

## Abbreviations

The following abbreviations are used in this manuscript:

TSK	Tight skin
WT	Wild type
CAF	Cancer-associated fibroblasts
TAM	Tumor-associated macrophages
ECM	Extracellular matrix
TCGB	Technology Center for Genomics & Bioinformatics
UCLA	University of California, Los Angeles
UMI	Unique molecular identifier
FDR	False Discovery Rate
UMAP	Uniform manifold approximation and projection
TCGA	The Cancer Genome Atlas
AOCS	Australian Ovarian Cancer Study
