# Supplementary material for: Cancer Growth and Invasion Are Increased in the Tight Skin (TSK) Mouse
Source: Cancers (Basel). 2025 Sep 9;17(18):2943. doi: 10.3390/cancers17182943 (PMC12468543; doi:10.3390/cancers17182943)
Supplement: Supplementary file 1 [file cancers-17-02943-s001.zip › Suppl Figures Cancers revised.pdf]

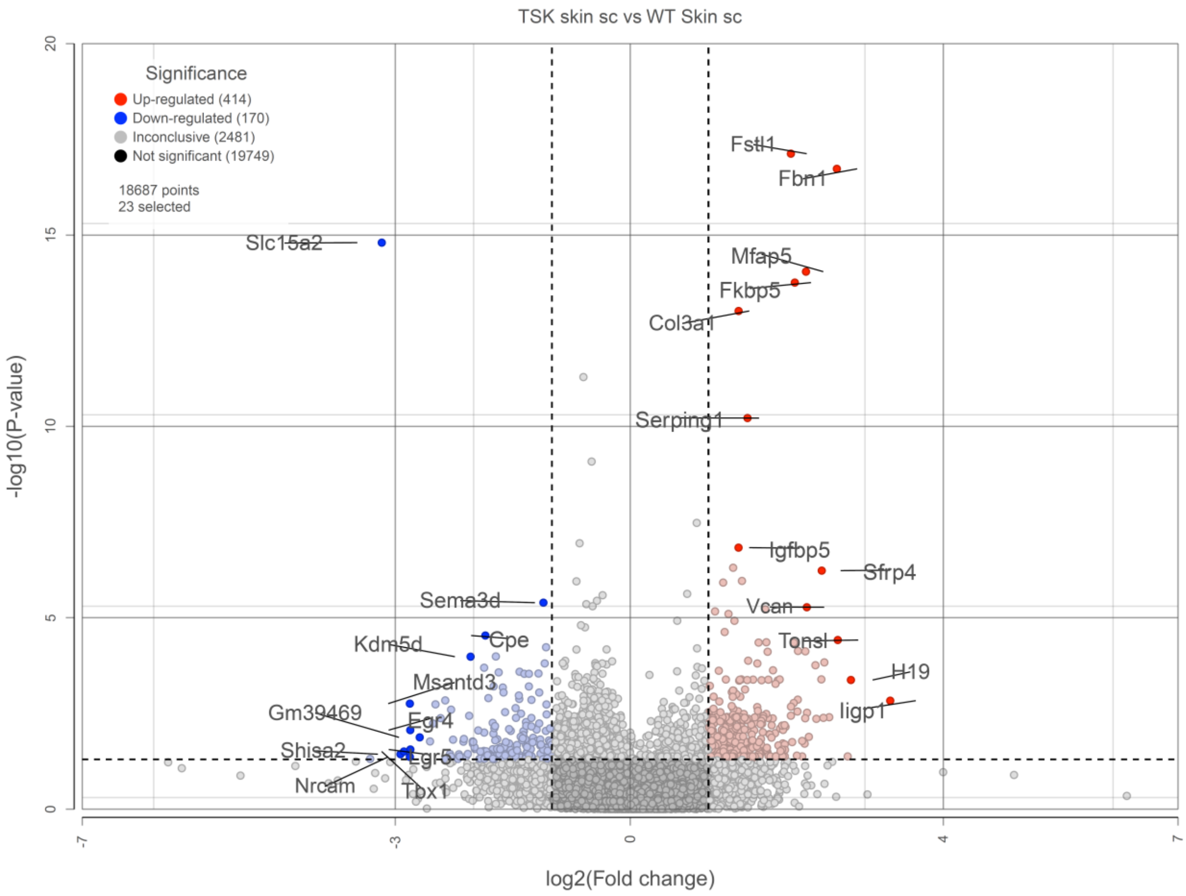

Figure S1. (Related to Fig.1e,f) Differentially expressed genes in keratinocytes from dorsal skins of WT and TSK mice. Volcano plot of differentially expressed genes in cell cluster identified as keratinocytes by single-cell RNA sequencing and graph-based clustering.

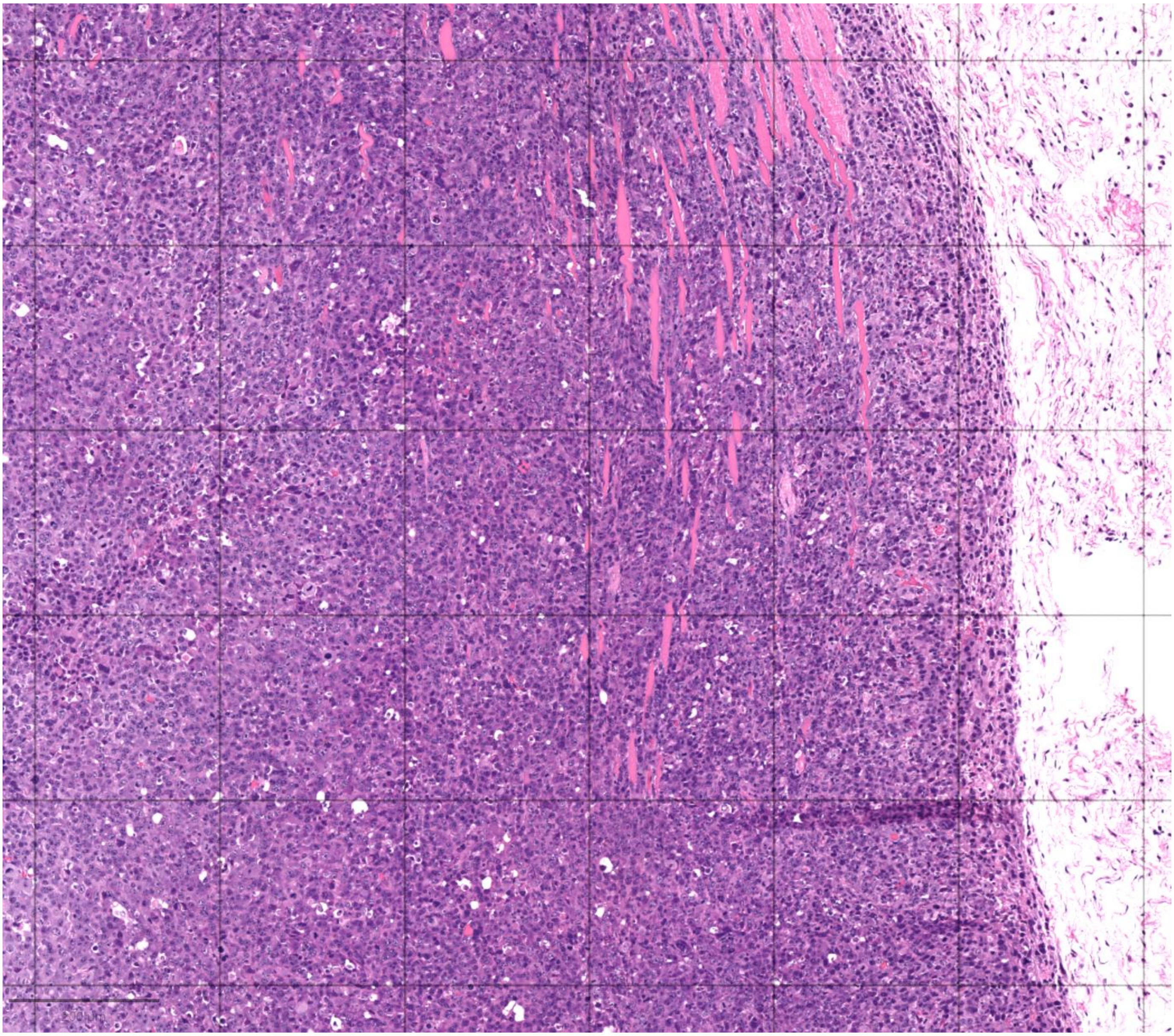

Figure S2. (Related to Fig. 2c,d) Quantification of tumor invasiveness: Tumor sections stained with H&E were digitized at 20x magnification using the Leica Aperio AT Turbo slide scanner. The digitized images were overlaid with 250 µm square tiles, and each tile was manually scored as positive if it contained muscle cells, adipose cells, or blood vessels encircled by cancer cells.

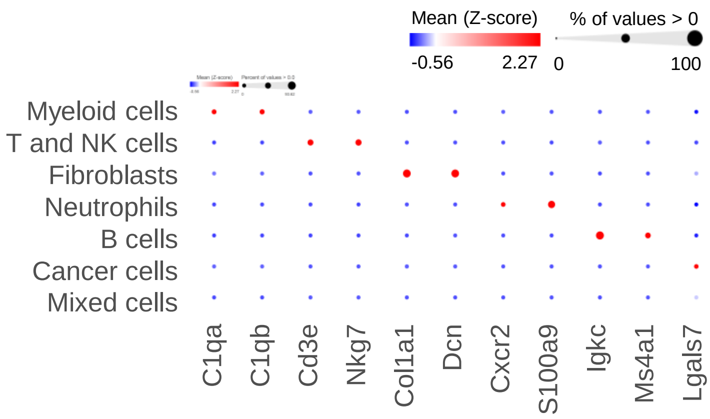

Figure S3. (Related to Fig. 2e,f) Expression of canonical cell type markers in subcutaneous melanoma B16F10 tumors analyzed by single-cell RNA sequencing. Dot plot showing the expression of representative cell-type specific markers across different cell clusters. The color intensity reflects the average gene expression, and the size indicates the percentage of cells expressing the gene within that cell type.

a

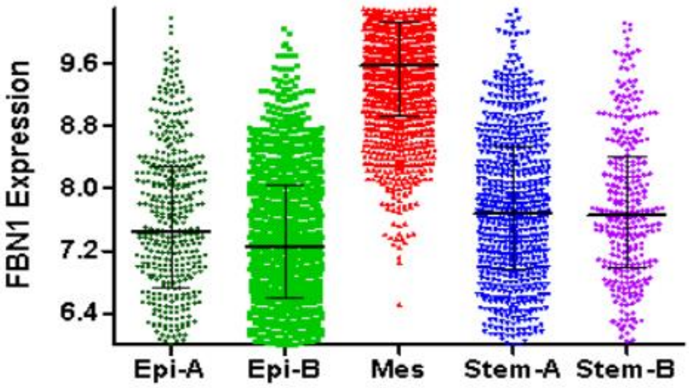

b

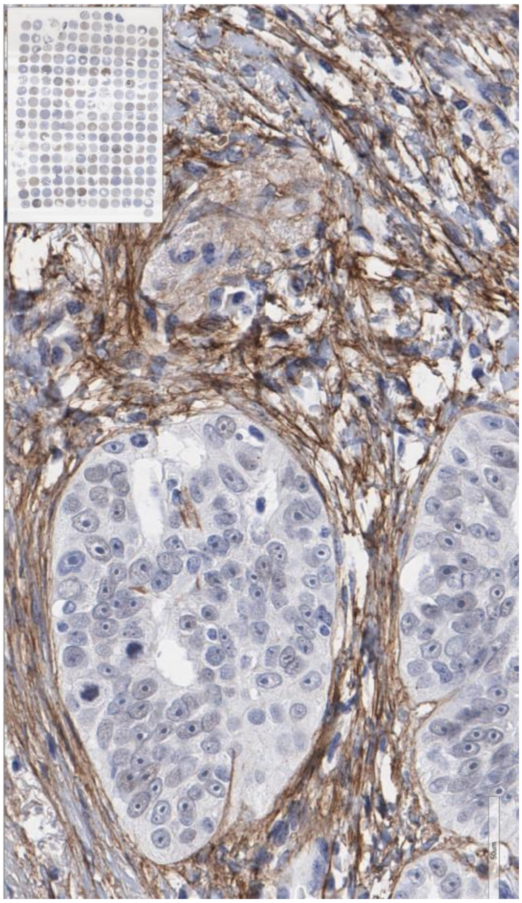

c

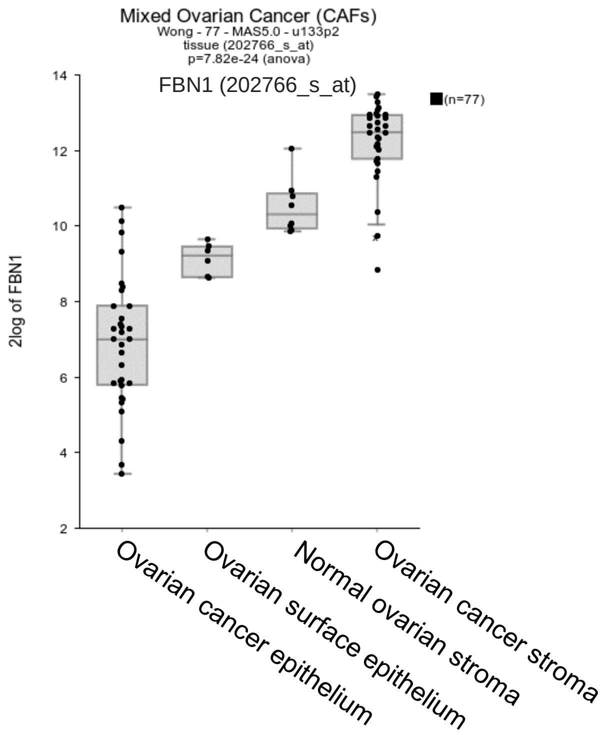

Tested combination	Welch p-value
Normal ovarian stroma vs Ovarian cancer stroma	9.11E-05
Normal ovarian stroma vs Ovarian surface epithelium	1.09E-03
Normal ovarian stroma vs Ovarian cancer epithelium	1.33E-09
Ovarian cancer stroma vs Ovarian surface epithelium	3.01E-10
Ovarian cancer stroma vs Ovarian cancer epithelium	2.85E-20
Ovarian surface epithelium vs Ovarian cancer epithelium	3.51E-07

Figure S4. Fibrillin expression in human ovarian cancer. (a) FBN1 expression in different molecular subtypes of ovarian cancer*. **(b)** Immunohistochemical detection of FBN1 in a tissue microarray of high-grade serous ovarian cancer. **(c)**, FBN1 RNA expression in laser capture microdissected epithelial and stromal cell types in the normal ovary and ovarian carcinoma.**

*http://csiovdb.mc.ntu.edu.tw/CSIOVDB.html; accessed 08/13/2023.

**https://hgserver2.amc.nl/cgi-bin/r2/main.cgi?openpage=login; accessed 08/13/2023.

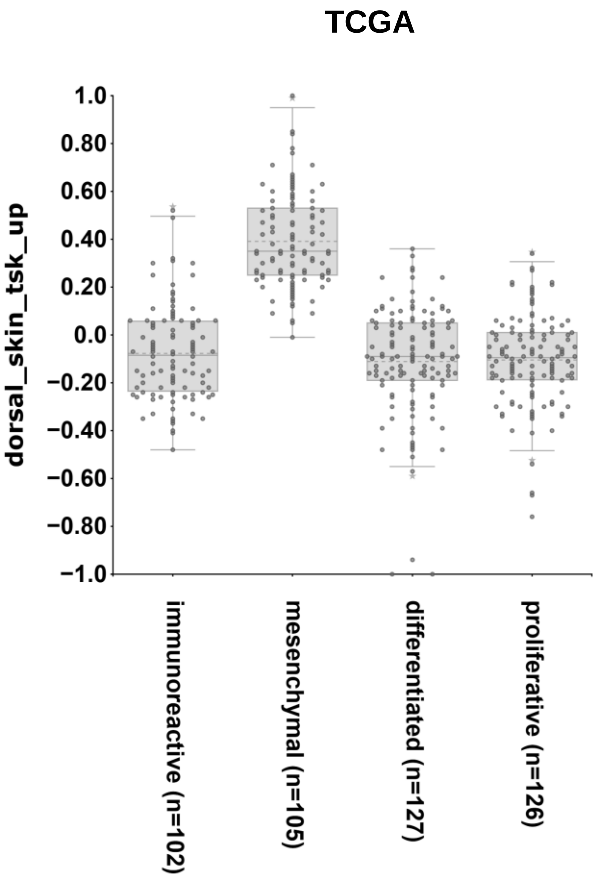

One Way Analysis of variance (ANOVA):

ANOVA	sum_square	df	mean_square	F	p-value
Between	19.551	3	6.517	156.593	**9.25e-70**
Within	18.977	456	0.042	-	-

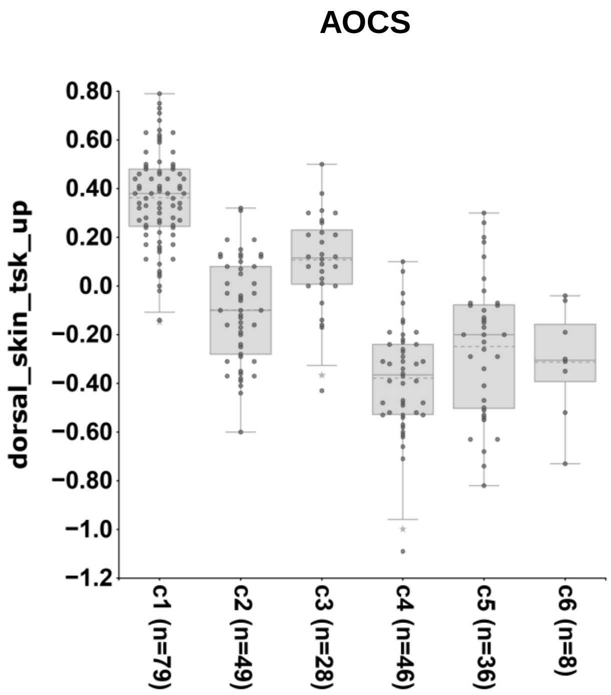

One Way Analysis of variance (ANOVA):

ANOVA	sum_square	df	mean_square	F	p-value
Between	20.754	5	4.151	86.726	**8.56e-52**
Within	11.487	240	0.048	-	-

Figure S5. Gene signature enriched in the skin of TSK mice is associated with the mesenchymal (c1) molecular subtype of ovarian cancer. Relative expression of the 185-gene signature upregulated in adult dorsal skin of TSK mice in TCGA and AOCS molecular subtypes of ovarian cancer. The plots were generated using R2 Genomics Analysis and Visualization Platform (https://hgserver2.amc.nl/cgi-bin/r2/main.cgi?open_page).

a

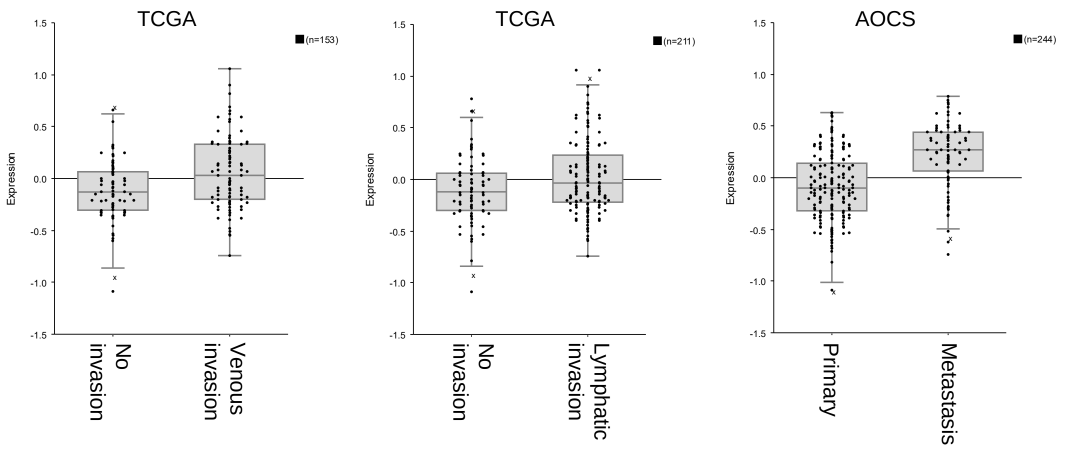

ANOVA	sum_square	df	mean_ square	F	p-value
Between	1.096	1	1.096	10.310	**1.62e-03**
Within	16.051	151	0.106	-	-

ANOVA	sum_square	df	mean_ square	F	p-value
Between	0.875	1	0.875	7.515	**6.65e-03**
Within	24.345	209	0.116	-	-

ANOVA	sum_square	df	mean_ square	F	p-value
Between	4.954	1	4.954	46.884	**6.16e-11**
Within	25.573	242	0.106	-	-

b

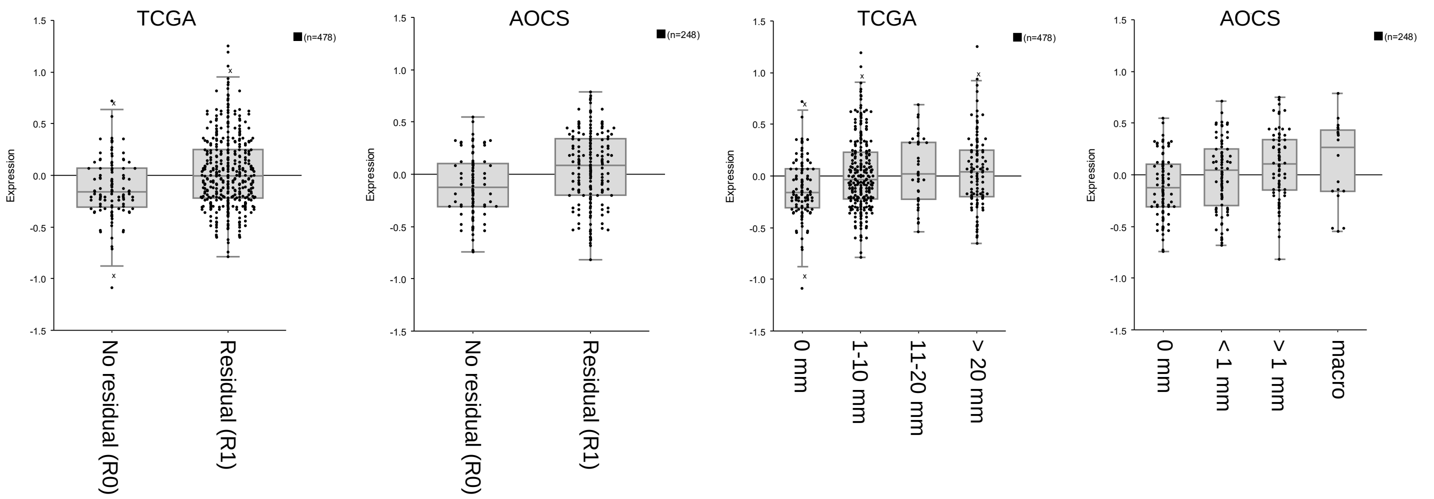

ANOVA	sum_square	df	mean_ square	F	p-value
Between	2.102	1	2.102	18.747	**1.82e-05**
Within	53.367	476	0.112	-	-

ANOVA	sum_square	df	mean_ square	F	p-value
Between	1.484	1	1.484	13.122	**3.54e-04**
Within	27.817	246	0.113	-	-

ANOVA	sum_square	df	mean_ square	F	p-value
Between	2.196	3	0.732	6.513	**2.53e-04**
Within	53.273	474	0.112	-	-

ANOVA	sum_square	df	mean_sq uare	F	p-value
Between	1.873	3	0.624	5.555	**1.05e-03**
Within	27.427	244	0.112	-	-

Figure S6. Gene signature enriched in the skin of TSK mice is associated with ovarian cancer invasion, metastasis, and suboptimal surgical debulking. **(a)** Relative expression of the 185-gene signature upregulated in adult dorsal skin of TSK mice in TCGA and AOCS ovarian cancer patients with different levels of lymphovascular invasion and site of tumor collection. Primary tumors were collected from the ovary, adnexa, or fallopian tube while metastases were collected from the upper abdomen. **(b)**, Relative expression of the 185-gene signature upregulated in adult dorsal skin of TSK mice in TCGA and AOCS high-grade serous ovarian cancer patients with different outcomes of primary debulking surgery. The plots were generated using R2 Genomics Analysis and Visualization Platform (https://hgserver2.amc.nl/cgi-bin/r2/main.cgi?open_page).

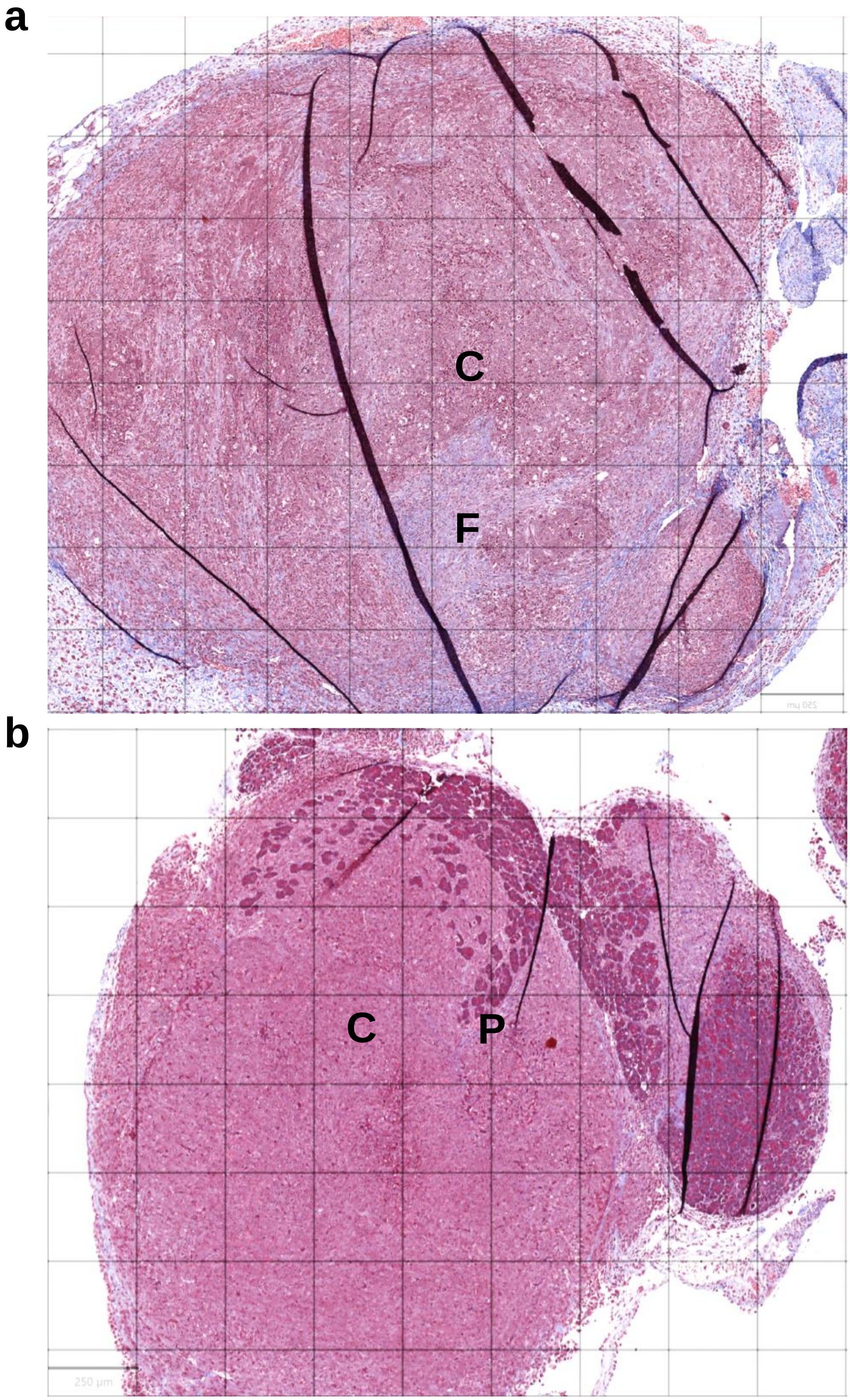

Figure S7. (Related to Fig. 4f and Fig. 5d) Quantification of tumor invasiveness: Tumor sections stained with Masson's trichrome were digitized at 20x magnification using the Leica Aperio AT Turbo slide scanner. The digitized images were overlaid with 250 µm square tiles, and each tile was manually scored as positive if it contained noncancer tissue encircled by cancer cells. **(a)** noncancer cells included fibroblasts, muscle cells, adipose cells, or blood vessels encircled by cancer cells. C, cancer; F, fibroblasts. **(b)**, noncancer cells included fibroblasts, muscle cells, adipose cells, blood vessels, and peritoneal organ tissues encircled by cancer cells. C, cancer; F, fibroblasts, P, pancreas.

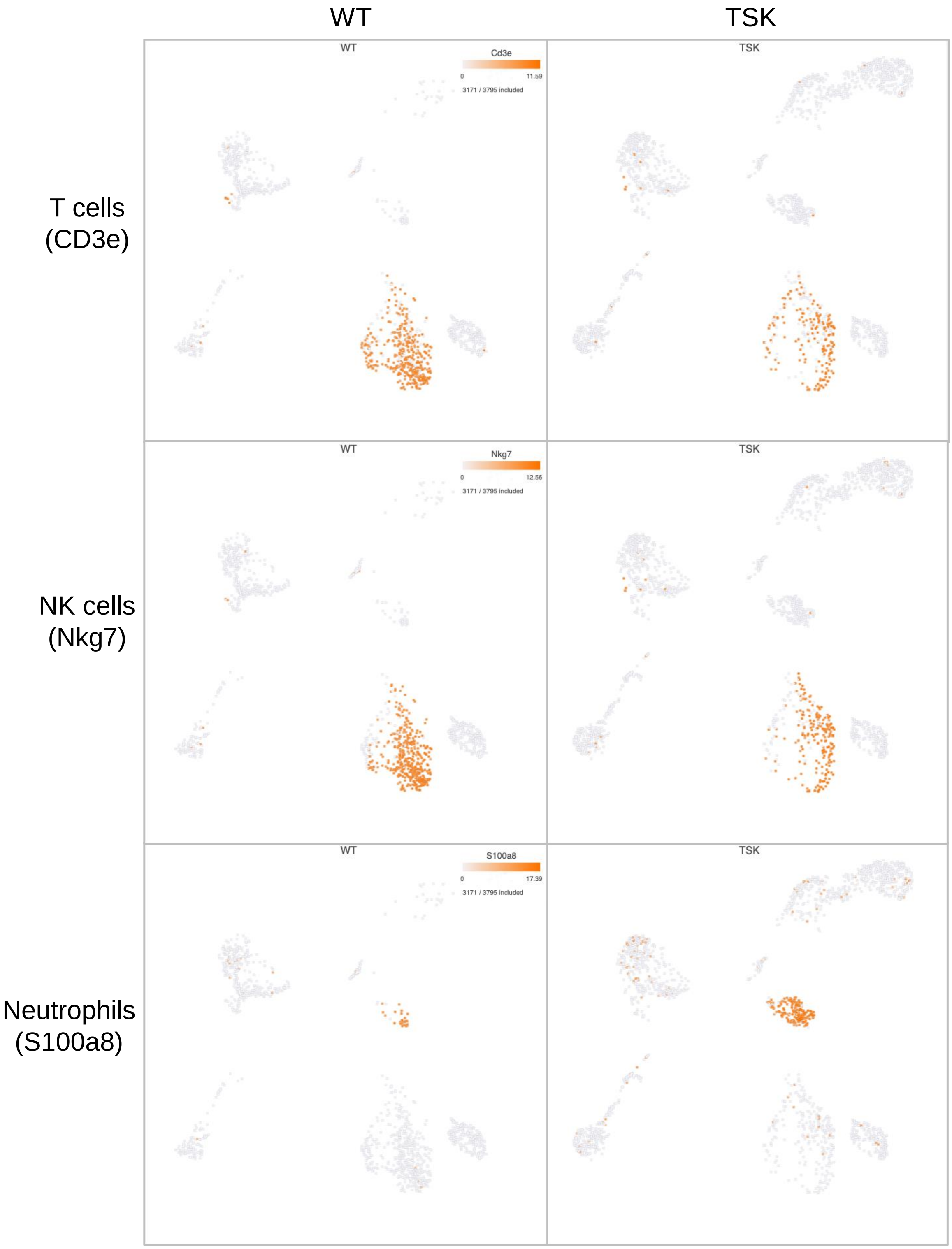

Figure S8. (Related to Fig. 6b) Ovarian tumors from WT mice are enriched for T cells and NK cells, while ovarian tumors from TSK mice are enriched for neutrophils. Single-cell RNA sequencing UMAP plot clusters overlaid with gene expression heat maps of transcripts that delineate T cells (Cd3e), NK cells (Nkg7), and neutrophils (S100a8). The color gradients indicate log2 expression. The clustering analysis and gene expression gradients were plotted using Partek software (Illumina).

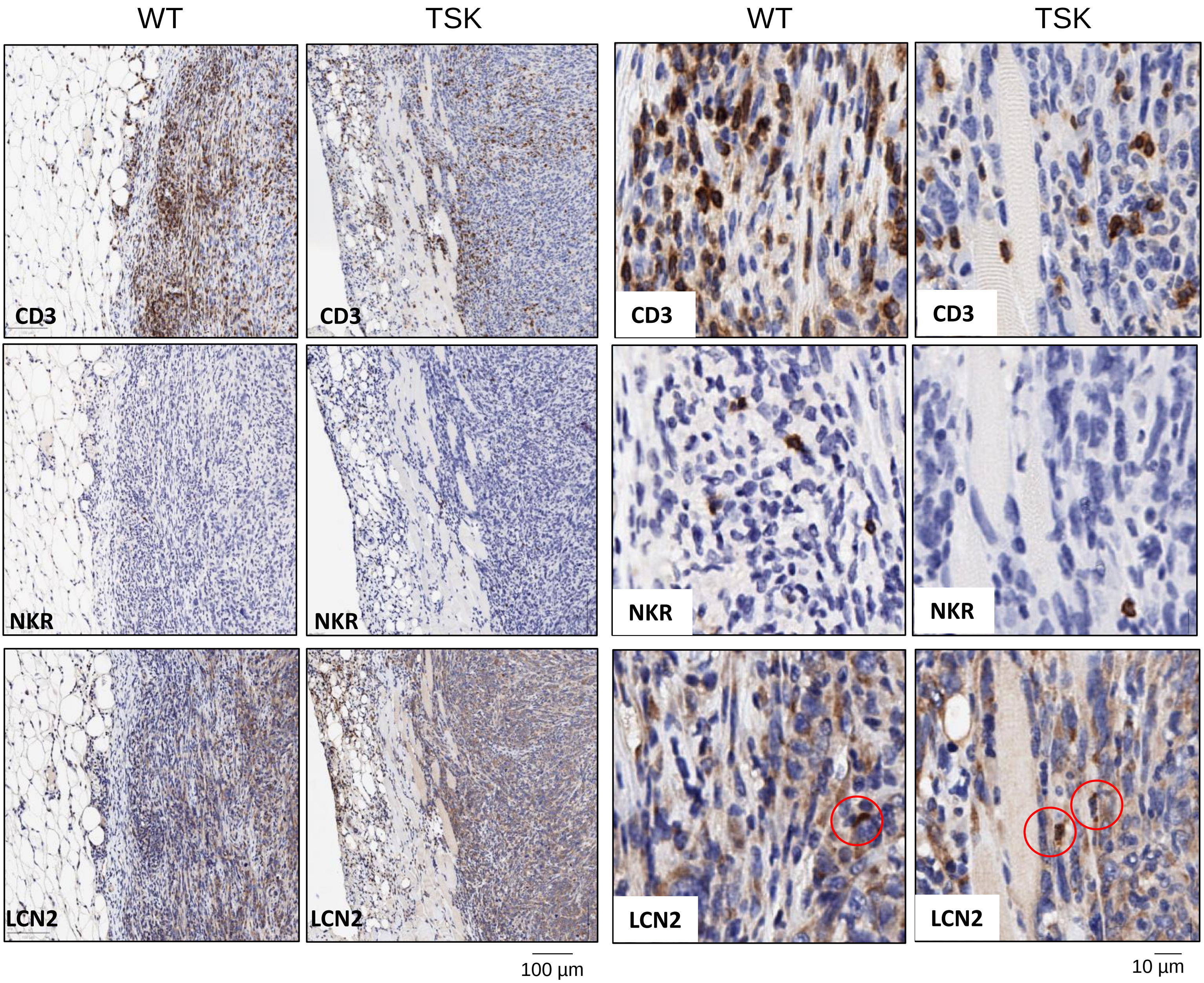

Figure S9. (Related to Fig. 6e) Immunohistochemical detection of T cells (CD3 antibody), NK cells (NKR-P1 antibody), and neutrophils (LCN2 antibody). Shown are representative consecutive sections of subcutaneous ovarian cancers in WT and TSK mice at low and high magnification. Red circles indicate LCN2 positive cells.

Melanoma B16-m10			
**Gene**	**P value**	**FDR step up**	**Fold change in TSK**
**Pf4**	0.28	0.99	1.57
**Retnla**	0.5	0.87	2.31

Breast Cancer EO771			
**Gene**	**P value**	**FDR step up**	**Fold change in TSK**
**Pf4**	0.38	0.60	1.30
**Retnla**	0.65	0.82	3.00

Figure S10 (Related to Fig 7c) Upregulation of Pf4 and Retnla expression in myeloid cells in melanoma and breast tumors in TSK mice. The tables show single-cell RNA sequencing data in myeloid cell clusters in B16-m10 melanoma and breast cancer EO771 breast cancer.
